# Mineral Content of Apple, Sour Cherry and Peach Pomace and the Impact of Their Application on Bakery Products

**DOI:** 10.3390/foods14183146

**Published:** 2025-09-09

**Authors:** Maria Bianca Mandache, Sina Cosmulescu

**Affiliations:** 1 Doctoral School of Plant and Animal Resources Engineering, Faculty of Horticulture, University of Craiova, A.I. Cuza Street, no. 13, 200585 Craiova, Romania; mandachemaria@yahoo.com; 2 Department of Horticulture and Food Science, Faculty of Horticulture, University of Craiova, A.I. Cuza Street, no. 13, 200585 Craiova, Romania

**Keywords:** apple pomace, sour cherry pomace, peach pomace, minerals

## Abstract

The aim of this paper was to analyze the mineral composition of pomace and bakery products supplemented with different weights (5%, 10% and 15%) of apple, sour cherry and peach pomace. The total mineral concentrations in pomace and bread were analyzed by inductively coupled plasma optical emission spectrometry. The analysis of mineral elements revealed that sour cherry pomace is a source of Ca (39.54 mg/100 g) and Cu (0.48 mg/100 g), and peach pomace of K (542.14 mg/100 g) and Mg (23.91 mg/100 g). In breads with the addition of sour cherry pomace, the highest concentrations of Ca (370.77 mg/100 g) and Mg (19.48 mg/100 g) were identified, while in bread with peach pomace, Cu (0.24 mg/100 g), Fe (0.92 mg/100 g), K (209.33 mg/100 g), Na (363.27 mg/100 g) and Zn (0.57 mg/100 g) predominated. Bread with apple pomace showed the highest content of Cr (0.016 mg/100 g) and Mn (0.53 mg/100 g). The results obtained attest that fruit pomace is a valuable ingredient, its micronutrient content having the ability to increase the nutritional value of food products.

## 1. Introduction

Fruit pomace (peel, pulp and seeds) can account for up to 50% of total fruit mass [1,2] and represents an important source of phytochemicals [3], vitamins and minerals [4]. Minerals such as calcium, iron, cooper, magnesium, manganese and zinc are essential components in human nutrition [5], playing a fundamental role in physiological and metabolic processes [6], including bone formation, the functioning of the muscular and nervous systems, regulation of the body’s water balance and support of the immune response [7]. A varied and balanced diet can generally ensure the optimal content of essential minerals for the body [8], but contemporary lifestyle and agricultural production methods have influenced this balance towards some of their insufficiencies [6]. Cereals occupy a central place in the diet of the population [9] worldwide, with bakery products being considered staple foods. Typically, white bread, obtained from refined wheat flour, is characterized by a low content of essential amino acids and minerals [10]. Mineral deficiency is associated with significant medical disorders [11]. Untreated, these deficiencies can lead to changes in the immune response, affect physical development, cognitive function and vulnerability to diseases [12].

Micronutrient deficiencies, also known as “hidden hunger”, are more widespread than their excess [13,14]. Deficiencies of iron, magnesium, zinc and calcium affect billions of people globally, leading to a negative impact on public health and the economy, in particular, iodine, iron, zinc, fluoride and selenium deficiencies contribute to the large number of nutritional diseases [15]. Thus, to prevent diseases caused by this deficiency, foods can be fortified with essential micronutrients [15], with fruit pomace being a natural source of cooper, magnesium, iron, potassium, calcium, etc. For example, the study conducted by Cacak-Pietrzak et al. [16] showed that bread enriched with aronia pomace had a higher mineral content, demonstrating the potential of pomace to improve the nutritional value of bakery products.

Numerous studies have reported the presence of phenolic compounds, dietary fibers, vitamins and minerals in pomace obtained from various fruits, highlighting their antioxidant role and nutritional contribution [16,17,18]. These bioactive compounds, such as flavonoids, polyphenols and anthocyanins, have been associated with cellular protection, reduction in oxidative stress, modulation of inflammation, maintenance of cardiovascular and metabolic health, regulation of immunity and prevention of DNA damage [19,20].

The novelty of the present study lies in the comparative analysis of the mineral composition of pomace obtained from three different fruits and in determining its applicability in bakery products—an aspect that has been addressed only to a limited extent. In this way, the study can make an important contribution both to the sustainable valorization of by-products from the fruit processing industry and to the development of functional foods with benefits for consumer health.

The aim of this study was to investigate the mineral content of apple, sour cherry and peach pomace, as well as to evaluate the influence of its incorporation, in varying concentrations, on the mineral profile of processed bakery products.

## 2. Materials and Methods

### 2.1. Materials

The pomace of apples, cherries and peaches, resulting from cold pressing of the fruits, was collected from two juice processors: SC Ancorvita SRL (Craiova, Romania) and the Rodiana Fruit Farm SRL ( Sibiu, Romania). Subsequently, the fruit pomace was dried at a temperature of 50 °C and 60 °C until the moisture level reached a constant level, then ground with an electric grinder and stored in hermetically sealed glass containers at a temperature of 4 °C. The obtained powder was used for the partial replacement of wheat flour in bakery products.

### 2.2. Technological Method of Obtaining Breads

The ingredients used to obtain the control bread included: wheat flour type 000 (250 g), baker’s yeast (*Saccharomyces cerevisiae*) Pakmaya (4 g), salt (iodized sodium chloride) (4 g) and drinking water (150 mL). To make fortified breads, part of the white wheat flour (5%, 10% and 15%) was replaced with apple, sour cherry and peach pomace.

The bakery products were prepared in a bakery, following a technological process that provided kneading (6 min), fermentation (15 min) and the division and shaping of the dough into equal-sized pieces. Subsequently, the dough was fermented at a temperature of 35 °C (60 min for the control bread and 90 min for the one with the addition of pomace) and a humidity of 75%, notched and baked at 250 °C for 25 min in a hearth oven. To achieve dough with optimal volume and consistency, the fermentation of bread supplemented with pomace was extended compared to the control bread, as the fibers and bioactive compounds in the pomace slow down yeast activity.

### 2.3. Sample Preparation

For the determination of metal content, between 4.8 g and 5.1 g of each type of pomace (apple, sour cherry and peach) and of bakery products from each assortment, supplemented with these pomaces, were cold-disaggregated for 6 weeks in 15 cm^3^ of spectrally pure nitric acid (Merck-Sigma-Aldrich, Darmstadt, Germany). Subsequently, the solutions were filtered and the final volume was completed with ultrapure water in 25 cm^3^ volumetric flasks.

### 2.4. Elemental Analysis

The concentrations of elements (Ca, Cr, Cu, Fe, K, Mg, Mn, Na, Zn) were determined with the spectrometer (ICP OES Thermo Scientific model iCAP 6000, Thermo Fisher Scientific, Waltham, MA, USA) according to the methodology described by Dróżdż et al. [21]. The working parameters of the spectrometer were as follows: Power 1.15 kW, auxiliary Ar gas flow rate 0.2 L/min, nebulizing Ar gas flow rate 0.42 L/min, coolant gas flow rate of 12 L/min. For each element analyzed, the spectral lines used were Ca 422.673 nm; Cr 283.563 nm; Cu 224.700 nm; Fe 259.940 nm; K 766.490 nm; Mg 280.270 nm; Mn 257.610 nm; Na 589.592 nm; Zn 213.856 nm.

### 2.5. Statistical Analysis

Each sample was performed in triplicate, and the values obtained were used to calculate the arithmetic mean and standard deviation (X ± SD), using Microsoft Excel 2010 software. Statistical analysis was performed using IBM SPSS Statistics 26 software and included two-factor ANOVA and Duncan multiple range tests (*p* < 0.05).

## 3. Results and Discussions

### 3.1. Mineral Content of Apple, Sour Cherry and Peach Pomace

The results obtained in the present study highlight the mineral composition of apple, sour cherry and peach pomace, emphasizing its potential for use in order to compensate for mineral deficiencies. The following mineral elements were quantified in the three types of pomace: Ca (calcium), Cr (chromium), Cu (copper), Fe (iron), K (potassium), Mg (magnesium), Mn (manganese), Na (sodium) and Zn (zinc). According to the data represented in Table 1, in sour cherry pomace the predominant mineral elements were calcium (39.54 mg/100 g), copper (0.48 mg/100 g) and iron (0.97 mg/100 g). Higher concentrations of chromium (0.0093 mg/100 g), potassium (542.14 mg/100 g), magnesium (23.91 mg/100 g), manganese (0.27 mg/100 g), sodium (2.56 mg/100 g) and zinc (0.33 mg/100 g) were identified in peach pomace.

Comparatively, in the study conducted by Singh and Kulshrestha. [22], the concentration of calcium (33.33 mg/100 g) and iron (2.512 mg/100 g) was lower in peach pomace. In apple pomace, although lower concentrations of mineral elements were recorded compared to sour cherry and peach pomace, a high potassium content (209.63 mg/100 g) was observed. In accordance with the results obtained, Kruczek et al. [23] and Neshovska [24] confirm the predominant presence of potassium in apple pomace.

Comparatively, Antonic et al. [25] identified higher concentrations of sodium (2–200 mg/100 g), potassium (449 mg/100 g), calcium (50–150 mg/100 g), magnesium (20–45 mg/100 g), iron (2.4–23 mg/100 g), zinc (0.22–1.5 mg/100 g) and manganese (0.61–0.9 mg/100 g) in apple pomace. Variability in mineral content is also reported in the study by Skinner et al. [26], where concentrations of 185.3 mg/100 g sodium, 398.4–880.2 mg/100 g potassium, 55.6–92.7 mg/100 g calcium, 18.5–333.5 mg/100 g magnesium, 2.9–3.5 mg/100 g iron, 1.4 mg/100 g zinc, 0.1 mg/100 g copper and 0.4–0.8 mg/100 g manganese were identified in apple pomace.

The mineral content of pomace varies significantly depending on the species, this being the main determining factor, since each type—apple, sour cherry, or peach—has a distinct biochemical profile that influences the final composition in potassium, calcium, phosphorus and other essential micronutrients. The variations found in previous studies have been attributed to a series of factors such as variety, climate, soil, processing conditions and extraction methods [27]. Following the results presented, it can be stated that apple, sour cherry and peach pomace can be considered an important mineral resource, with significant potential in the development of food products with functional properties.

### 3.2. Mineral Content in Breads Enriched with Apple, Cherry and Peach Pomace

Supplementation of wheat breads with fruit pomace is often associated with increased mineral concentrations [28]. The results presented in Table 2 reflect the influence of pomace application on the mineral content of breads supplemented with progressive amounts (5, 10 and 15%) of apple, cherry and peach pomace. Depending on the type and weight of pomace applied, the content of mineral elements fluctuated significantly (*p* < 0.05). Of all the samples analyzed, the lowest calcium concentration was identified in the control bread (73.13 mg/100 g). Its content increased in accordance with the percentage of peach pomace applied (5–15%), from 19.70 to 23.75 mg/100 g.

In contrast, in breads with apple pomace the trend was opposite, the maximum level being identified at an addition of 5% pomace (225 mg/100 g). In breads with sour cherry pomace, an oscillating evolution was observed, the maximum concentration being recorded at an addition of 10% pomace (370.77 mg/100 g). Overall, breads with the addition of sour cherry pomace recorded the highest calcium concentrations, followed by those with apple and peach pomace, this order reflecting the natural concentration of calcium in the fruit and the degree of retention in post-processing residues.

Higher concentrations of certain minerals at a low pomace addition (5%), followed by a decrease at higher substitution levels (10–15%), may indicate that, at low levels, pomace promotes the release and stabilization of minerals. In contrast, at higher concentrations, some compounds such as oxalic acid and polyphenols can form insoluble complexes, reducing the amount of bioavailable minerals [29]. Additionally, increased pomace addition can alter the dough structure and the distribution of minerals, contributing to this nonlinear variation.

The supplementation of bakery products with pomace did not lead to an increase in chromium content, the control sample recording the highest level (0.016 mg/100 g). In fortified breads, the highest chromium levels were observed with the addition of 5% apple pomace (0.015 mg/100 g), 10% cherry pomace (0.013 mg/100 g) and 5 and 15% peach pomace (0.013 mg/100 g). In general, chromium from pomace is retained very well in bread after baking due to its thermal stability, the differences from the control occur due to the complex composition of pomace—rich in fiber, minerals, phenolic compounds and organic acids.

The increase in the pomace weight (5–15%) generally determined an evolutionary increase in copper concentrations. Depending on the type of pomace used, the content showed significant variations, both at a minimum level (0.14–0.22 mg/100 g) and at a maximum addition level (0.17–0.24 mg/100 g). The only distinction was found in the control sample which presented a higher content (0.15 mg/100 g), compared to the sample which included a concentration of 5% apple pomace. The increase in the amount of pomace in the dough leads to an additional copper intake, but the variations are not linear, due to the uneven distribution of copper and technological losses [30]. Regardless of the proportion of pomace used, formulations with peach pomace showed the highest copper content, compared to those with sour cherry and apple pomace.

In the bread making process, the integration of different weights of fruit pomace generated a significant increase in iron content. In relation to the control sample (0.50 mg/100 g), an increase in levels was identified depending on the amount and type of pomace applied, the range of oscillations recording higher values at an addition of 15% (0.56, 0.63 and 0.92 mg/100 g). Of all the samples analyzed, peach pomace recorded the highest iron content, followed by sour cherry and apple pomace. In conclusion, the supplementation of bread with fruit pomace determined a significant increase in iron content, this being directly proportional to the percentage of pomace added and influenced by the type of fruit used.

Depending on the type and concentration of pomace applied, the level of magnesium fluctuated significantly in all the samples analyzed. In breads with the addition of apple pomace (5–15%), its content registered a linear decrease (18.75–17.36 mg/100 g), while in breads fortified with sour cherry pomace it generated an upward expansion of the content (17.55–19.48 mg/100 g). In the case of breads enriched with peach pomace, the addition of 10% led to the lowest content (17.51 mg/100 g), and the addition of 15% to the highest (18.36 mg/100 g). The level of potassium and magnesium varied due to the differences in their concentrations in the types of pomace used and the way in which they influence the baking process.

Regarding the amount of manganese, the control sample showed a higher level (0.43 mg/100 g), compared to the samples with peach pomace (0.35–0.38 mg/100 g), but lower than those with apple pomace (0.49–0.53 mg/100 g) and sour cherry (0.46–0.47 mg/100 g). The growth trend was not linear in relation to the share of integrated pomace, the breads with apple pomace, registering a directly proportional decrease. The amount of manganese decreased in the breads with apple pomace, probably due to its low manganese content or technological factors that reduced its retention in the final product.

Sodium generally showed a downward trend compared to the control sample (362.89 mg/100 g). The values identified in bread supplemented with 5% apple, sour cherry and peach pomace were between 198.96, 258.23 and 97.81 mg/100 g, respectively, while in breads with 15% pomace the level was 269.51, 266.17 and 363.27 mg/100 g. The amount of zinc showed an uneven evolution in relation to the percentage of pomace used. The lowest value was detected in breads with sour cherry pomace (0.42–0.47 mg/100 g), followed by the control sample (0.48 mg/100 g), breads fortified with apple pomace (0.45–0.55 mg/100 g) and those with peach pomace (0.51–0.57 mg/100 g).

While copper, iron and potassium showed increasing trends, magnesium, calcium, chromium, sodium and zinc showed a disproportionate variation in relation to the applied pomace concentration. In the study conducted by Stanciu et al. [31], the supplementation of breads with different varieties and concentrations of sea buckthorn led to a decrease in zinc content. The same authors found that the increase in manganese content occurred only in samples that included sea buckthorn pomace powder from the Clara (6–8%) and Mara (8–10%) varieties. In comparison, Cantero et al. [32] reported that the addition of 8% apple pomace to gluten-free bread caused an increase in the content of Cu, Mg, Zn, Fe, Ca and Mn, from initially non-existent values.

This aspect can be attributed to the complexity of the behavior of minerals in food matrices [33]. Minerals in the structure of foods are found in the form of free ions, complexes or compounds [34]. Free ions show increased reactivity due to their high solubility and ability to interact with biochemical components of foods through covalent and electrostatic bonds [34]. Also, during food processing and storage, minerals undergo complex changes due to their varied chemical forms, relatively low and diverse concentrations [35].

### 3.3. Covering the Daily Mineral Requirements

Fortifying bread with fruit pomace can become a viable solution to increase mineral composition and nutritional value. Table 3 shows the average mineral content recorded in breads with added apple, cherry and peach pomace compared to the DRI (Recommended dietary allowances and adequate intake). Following the analysis of the presented data, it can be emphasized that a 50 g portion (according to Reference amounts customarily consumed) of bread with added fruit pomace covers the macroelement and microelement requirement in a relatively high percentage. Bread is not considered an important source of calcium, but the fortification process can significantly increase the content of this essential mineral [28]. On average, the consumption of 50 g of bread with added apple, cherry and peach pomace brought a calcium intake ranging between 1.04 and 16.03%. Calcium is an essential mineral involved in the formation of bones and teeth, the blood clotting process and muscle contraction [36].

On average, a 50 g portion of bread supplemented with pomace provided a chromium content higher than the other elements analyzed, covering the daily requirement by 17.14–18.57%. Chromium plays an important role in regulating cardiovascular risk factors through its effect on insulin concentration and activity [37]. Consumption of a 50 g portion of bread provided, on average, the copper and iron requirements by 8.33–12.22% and 3.5–5.31%, respectively. Copper plays an essential role in the synthesis of collagen and elastin and in the fixation of iron in hemoglobin [38], and iron is involved in the synthesis of hemoglobin and the maintenance of normal muscle function [38].

The results of this study show that breads with added pomace provide between 2.12 and 2.22% of the indicated magnesium requirement. Magnesium contributes to the transmission of neuromuscular impulses, facilitating muscle relaxation, and is also a structural part of ribosomes and is involved in protein synthesis [39]. The samples supplemented with pomace contributed to a high extent to covering the manganese requirement (7.83–11.09%). Manganese, an essential trace element, influences the metabolism of amino acids, cholesterol and carbohydrates and contributes to the development of bone tissue [40]. The consumption of 50 g of bread with the addition of pomace brought a potassium intake that ranged between 1.98 and 2.40%. An optimal level of potassium in the diet is fundamental for the normal functioning of the muscles, the regulation of water and acid-base balance and the maintenance of osmotic pressure in the body [41]. On average, the consumption of 50 g of bread supplemented with pomace provided between 6.36 and 8.73% sodium. Sodium plays an important role in maintaining acid-base balance, muscle contraction and nerve impulse transmission [36]. Fortified bread covered 2–2.45% of the zinc requirement. Zinc contributes to increasing the body’s resistance to infections and is involved in the synthesis and breakdown of carbohydrates, proteins and fats [28].

Therefore, the inclusion of fruit pomace in the recipe for manufacturing bakery products may be promising for increasing the proportion of minerals in the human diet.

## 4. Conclusions

The results show that apple, sour cherry and peach pomaces can be successfully incorporated into bakery products, representing an important source of minerals. Comparative analysis highlighted differences between the types of fruit, suggesting that the choice of raw material can be adapted according to specific nutritional needs. In relation to the recommended dietary allowances and adequate intake values for microelements, enriched breads contribute significantly to the intake of calcium, chromium, copper and sodium, thereby enhancing the functional profile of the products.

This innovative use supports current trends in the food industry, focusing both on the development of functional products and the sustainable valorization of agro-industrial by-products. Moreover, the study provides a foundation for future research on the impact of pomace on the sensory functional, and shelf-life properties of the final products.

## Figures and Tables

**Table 1:** Mineral element content of apple, sour cherry and peach pomace.

Sample	Apple Pomace	Sour Cherry Pomace	Peach Pomace
Calcium (mg/100 g)	10.1100 ± 0.0100 ^c^	39.5400 ± 0.0900 ^a^	25.0000 ± 0.0300 ^b^
Chromium (mg/100 g)	0.0065 ± 0.0001 ^c^	0.0085 ± 0.0001 ^b^	0.0093 ± 0.0005 ^a^
Copper (mg/100 g)	0.1200 ± 0.0001 ^c^	0.4800 ± 0.0004 ^a^	0.2600 ± 0.0003 ^b^
Iron (mg/100 g)	0.3300 ± 0.0004 ^c^	0.9700 ± 0.0030 ^a^	0.8200 ± 0.0010 ^b^
Potassium (mg/100 g)	209.6300 ± 0.4900 ^c^	327.3400 ± 0.7100 ^b^	542.1400 ± 0.9000 ^a^
Magnesium (mg/100 g)	8.8800 ± 0.0100 ^c^	19.8100 ± 0.0150 ^b^	23.9100 ± 0.0300 ^a^
Manganese (mg/100 g)	0.1600 ± 0.0001 ^b^	0.2300 ± 0.0007 ^a^	0.2700 ± 0.0003 ^a^
Sodium (mg/100 g)	1.9600 ± 0.0001 ^c^	2.1800 ± 0.0100 ^b^	2.5600 ± 0.0100 ^a^
Zinc (mg/100 g)	0.0900 ± 0.0006 ^b^	0.1400 ± 0.0009 ^b^	0.33 ± 0.0002 ^a^

X = mean; SD = standard deviation (n = 3). Distinct letters indicate statistically significant differences (Duncan’s multiple range test, *p* < 0.05).

**Table 2:** Mineral composition of breads with added apple, sour cherry and peach pomace.

	Mineral Content								
Sample	Ca (mg/100 g)	Cr (mg/100 g)	Cu (mg/100 g)	Fe (mg/100 g)	K (mg/100 g)	Mg (mg/100 g)	Mn (mg/100 g)	Na (mg/100 g)	Zn (mg/100 g)
Control sample	73.13 ± 0.1 ^g^	0.016 ± 0.0008 ^a^	0.15 ± 0.0001 ^de^	0.50 ± 0.0001 ^e^	91.73 ± 0.18 ^j^	16.46 ± 0.05 ^h^	0.43 ± 0.0008 ^e^	362.89 ± 0.81 ^b^	0.48 ± 0.0001 ^cd^
Pm 5%	225.30 ± 0.21 ^c^	0.015 ± 0.0001 ^ab^	0.14 ± 0.0005 ^e^	0.56 ± 0.0004 ^d^	119.38 ± 0.01 ^i^	18.75 ± 0.07 ^c^	0.53 ± 0.003 ^a^	269.51 ± 0.30 ^c^	0.50 ± 0.001 ^bc^
Pm 10%	212.25 ± 0.80 ^e^	0.011 ± 0.0008 ^b^	0.15 ± 0.0003 ^de^	0.56 ± 0.0001 ^d^	132.74 ± 0.18 ^f^	17.55 ± 0.03 ^e^	0.52 ± 0.0003 ^a^	198.96 ± 0.10 ^h^	0.55 ± 0.05 ^a^
Pm 15%	166.92 ± 0.70 ^f^	0.012 ± 0.0001 ^ab^	0.17 ± 0.0002 ^cde^	0.57 ± 0.001 ^d^	151.00 ± 0.18 ^c^	17.36 ± 0.04 ^g^	0.49 ± 0.001 ^b^	244.58 ± 0.30 ^g^	0.45 ± 0.0003 ^fg^
Pv 5%	221.22 ± 0.40 ^d^	0.012 ± 0.0001 ^ab^	0.17 ± 0.0003 ^cde^	0.57 ± 0.0007 ^d^	119.73 ± 0.14 ^h^	17.55 ± 0.03 ^e^	0.46 ± 0.0006 ^d^	266.17 ± 0.60 ^d^	0.42 ± 0.0003 ^h^
Pv 10%	370.77 ± 0.41 ^a^	0.013 ± 0.0002 ^ab^	0.19 ± 0.0001 ^bcd^	0.62 ± 0.0004 ^c^	145.25 ± 0.08 ^e^	19.02 ± 0.0002 ^b^	0.47 ± 0.0002 ^cd^	258.23 ± 0.33 ^f^	0.47 ± 0.0005 ^de^
Pv 15%	369.56 ± 0.10 ^b^	0.012 ± 0.0001 ^ab^	0.22 ± 0.0001 ^ab^	0.63 ± 0.001 ^c^	168.40 ± 0.30 ^b^	19.48 ± 0.04 ^a^	0.47 ± 0.001 ^cd^	261.38 ± 0.75 ^e^	0.44 ± 0.0002 ^gh^
Pp 5%	19.70 ± 0.02 ^i^	0.013 ± 0.0007 ^ab^	0.22 ± 0.0001 ^ab^	0.83 ± 0.0009 ^b^	150.26 ± 0.16 ^d^	17.53 ± 0.05 ^ef^	0.36 ± 0.0003 ^fg^	97.81 ± 0.14 ^j^	0.56 ± 0.0003 ^a^
Pp 10%	18.92 ± 0.04 ^j^	0.012 ± 0.0007 ^ab^	0.21 ± 0.0001 ^abc^	0.80 ± 0.002 ^b^	130.62 ± 0.32 ^g^	17.51 ± 0.02 ^f^	0.38 ± 0.01 ^f^	111.39 ± 0.24 ^i^	0.57 ± 0.0004 ^a^
Pp 15%	23.75 ± 0.05 ^h^	0.013 ± 0.0008 ^ab^	0.24 ± 0.0001 ^a^	0.92 ± 0.001 ^a^	209.33 ± 0.37 ^a^	18.36 ± 0.06 d	0.35 ± 0.0009 ^g^	363.27 ± 0.60 ^a^	0.51 ± 0.0001 ^b^

Ca-calcium, Cr-chromium, Cu-copper, Fe-iron, K-potassium, Mg-magnesium, Mn-manganese, Na-sodium, Zn-zinc; Pm 5, 10, 15%-bread with 5, 10, 15% added apple pomace; Pv 5, 10, 15%-bread with 5, 10, 15% added sour cherry pomace; Pp 5, 10, 15%-bread with 5, 10, 15% added peach pomace; X = mean; SD = standard deviation (n = 3). Distinct letters indicate statistically significant differences (Duncan’s multiple range test, *p* < 0.05).

**Table 3:** Reference quantities of minerals typically consumed.

Nutrient	Recommended Dietary Allowances and Adequate Intake		The Average Content in Breads with Added Apple Pomace	The Average Content in Breads with Added Sour Cherry Pomace	The Average Content in Breads with Added Peach Pomace
	Unit	DRI			
Calcium	mg	1000	201.49 ± 0.57	320.52 ± 0.30	20.79 ± 0.04
Chromium	mg	0.035	0.013 ± 0.0003	0.012 ± 0.0001	0.013 ± 0.0007
Copper	mg	0.9	0.15 ± 0.0003	0.19 ± 0.0001	0.22 ± 0.0001
Iron	mg	8	0.56 ± 0.0005	0.61 ± 0.0007	0.85 ± 0.001
Magnesium	mg	420	17.89 ± 0.05	18.68 ± 0.02	17.8 ± 0.04
Manganese	mg	2.3	0.51 ± 0.001	0.47 ± 0.0006	0.36 ± 0.004
Potassium	mg	3400	134.37 ± 0.12	144.46 ± 0.17	163.40 ± 0.28
Sodium	mg	1500	237.68 ± 0.23	261.93 ± 0.56	190.82 ± 0.33
Zinc	mg	11	0.5 ± 0.01	0.44 ± 0.0003	0.54 ± 0.0001

Source: https://ods.od.nih.gov/HealthInformation/nutrientrecommendations.aspx (accessed on 21 August 2025).

## Data Availability

The original contributions presented in this study are included in the article. Further inquiries can be directed to the corresponding author.

## Abbreviations

The following abbreviations are used in this manuscript:

PM	Bread with added apple pomace
PV	Bread with added sour cherry pomace
PP	Bread with added peach pomace

## References

[1] Raczkowska E., Serek P. (2024). Health-Promoting Properties and the Use of Fruit Pomace in the Food Industry—A Review. Nutrients.

[2] Martin-Dominguez V., Garcia-Montalvo J., Garcia-Martin A., Ladero M., Santos V.E. (2022). Fumaric Acid Production by *R. arrhizus* NRRL 1526 Using Apple Pomace Enzymatic Hydrolysates: Kinetic Modelling. Processes.

[3] Struck S., Rohm H. (2020). Fruit processing by-products as food ingredients. Valorization of Fruit Processing By-Products.

[4] Kruczek M., Drygaś B., Habryka C. (2016). Pomace in fruit industry and their contemporary potential application. World Sci. News.

[5] Weyh C., Krüger K., Peeling P., Castell L. (2022). The Role of Minerals in the Optimal Functioning of the Immune System. Nutrients.

[6] Alghamdi M., Gutierrez J., Komarnytsky S. (2023). Essential Minerals and Metabolic Adaptation of Immune Cells. Nutrients.

[7] Kim M.-H., Choi M.-K. (2013). Seven dietary minerals (Ca, P, Mg, Fe, Zn, Cu, and Mn) and their relationship with blood pressure and blood lipids in healthy adults with self-selected diet. Biol. Trace Elem. Res..

[8] Lukaski H.C. (2004). Vitamin and mineral status: Effects on physical performance. Nutrition.

[9] Dewettinck K., Van Bockstaele F., Kühne B., Van de Walle D., Courtens T.M., Gellynck X. (2008). Nutritional value of bread: Influence of processing, food interaction and consumer perception. J. Cereal Sci..

[10] Coțovanu I., Stroe S.-G., Ursachi F., Mironeasa S. (2023). Addition of Amaranth Flour of Different Particle Sizes at Established Doses in Wheat Flour to Achieve a Nutritional Improved Wheat Bread. Foods.

[11] Gharibzahedi S.M.T., Jafari S.M. (2017). The importance of minerals in human nutrition: Bioavailability, food fortification, processing effects and nanoencapsulation. Trends Food Sci. Technol..

[12] Razzaque M.S., Wimalawansa S.J. (2025). Minerals and Human Health: From Deficiency to Toxicity. Nutrients.

[13] Bailey R.L., West K.P., Black R.E. (2015). The Epidemiology of Global Micronutrient Deficiencies. Ann. Nutr. Metab..

[14] Vrech M., Ferruzzi A., Pietrobelli A. (2022). Effects of micronutrient and phytochemical supplementation on cardiovascular health in obese and overweight children: A narrative review. Curr. Opin. Clin. Nutr. Metab. Care.

[15] Awuchi C.G., Igwe V.S., Amagwula I.O., Echeta C.K. (2020). Health Benefits of Micronutrients (Vitamins and Minerals) and their Associated Deficiency Diseases: A Systematic Review. Int. J. Food Sci..

[16] Cacak-Pietrzak G., Dziki D., Gawlik-Dziki U., Parol-Nadłonek N., Kalisz S., Krajewska A., Stępniewska S. (2023). Wheat Bread Enriched with Black Chokeberry (*Aronia melanocarpa* L.) Pomace: Physicochemical Properties and Sensory Evaluation. Appl. Sci..

[17] Torbica A., Škrobot D., Hajnal E.J., Belović M., Zhang N. (2019). Sensory and physico-chemical properties of wholegrain wheat bread prepared with selected food by-products. LWT.

[18] Bhat I.M., Wani S.M., Mir S.A., Naseem Z. (2023). Effect of microwave-assisted vacuum and hot air oven drying methods on quality characteristics of apple pomace powder. Food Prod. Process. Nutr..

[19] Fernández-Fernández A.M., Dellacassa E., Nardin T., Larcher R., Gámbaro A., Medrano-Fernandez A., del Castillo M.D. (2021). In Vitro Bioaccessibility of Bioactive Compounds from Citrus Pomaces and Orange Pomace Biscuits. Molecules.

[20] Mohammadi N., Farrell M., O’Sullivan L., Langan A., Franchin M., Azevedo L., Granato D. (2024). Effectiveness of anthocyanin-containing foods and nutraceuticals in mitigating oxidative stress, inflammation, and cardiovascular health-related biomarkers: A systematic review of animal and human interventions. Food Funct..

[21] Dróżdż P., Šėžienė V., Pyrzynska K. (2018). Mineral Composition of Wild and Cultivated Blueberries. Biol. Trace Elem. Res..

[22] Singh S., Kulshrestha K. (2016). Peach juice and pomace powder; nutritive value and use of pomace powder in biscuits. Int. J. Food Sci. Technol..

[23] Kruczek M., Gumul D., Kačániová M., Ivanišhová E., Mareček J., Gambuś H. (2017). Industrial Apple Pomace By-Products as a Potential Source of Pro-Health Compounds in Functional Food. J. Microbiol. Biotechnol. Food Sci..

[24] Neshovska H. (2024). Determination of the Chemical and Mineral Composition of Apple Pomace in Relation to Its Utilisation as an Innovative Feed Raw Material. Tradit. Mod. Vet. Med..

[25] Antonic B., Jancikova S., Dordevic D., Tremlova B. (2020). Apple pomace as food fortification ingredient: A systematic review and meta-analysis. J. Food Sci..

[26] Skinner R.C., Gigliotti J.C., Ku K.-M., Tou J.C. (2018). A Comprehensive Analysis of the Composition, Health Benefits, and Safety of Apple Pomace. Nutr. Rev..

[27] Oney-Montalvo J., Uc-Varguez A., Ramírez-Rivera E., Ramírez-Sucre M., Rodríguez-Buenfil I. (2020). Influence of Soil Composition on the Profile and Content of Polyphenols in Habanero Peppers (*Capsicum chinense* Jacq.). Agronomy.

[28] Purkiewicz A., Gul F.H., Pietrzak-Fiećko R. (2024). The Utilization of Vegetable Powders for Bread Enrichment—The Effect on the Content of Selected Minerals, Total Phenolic and Flavonoid Content, and the Coverage of Daily Requirements in the Human Diet. Appl. Sci..

[29] Abera S., Yohannes W., Chandravanshi B.S. (2023). Effect of processing methods on antinutritional factors (oxalate, phytate, and tannin) and their interaction with minerals (calcium, iron, and zinc) in red, white, and black kidney beans. Int. J. Anal. Chem..

[30] García-Sartal C., del Carmen Barciela-Alonso M., Moreda-Piñeiro A., Bermejo-Barrera P. (2013). Study of cooking on the bioavailability of As, Co, Cr, Cu, Fe, Ni, Se and Zn from edible seaweed. Microchem. J..

[31] Stanciu I., Ungureanu E.L., Popa E.E., Geicu-Cristea M., Draghici M., Mitelut A.C., Mustatea G., Popa M.E. (2023). The Experimental Development of Bread with Enriched Nutritional Properties Using Organic Sea Buckthorn Pomace. Appl. Sci..

[32] Cantero L., Salmerón J., Miranda J., Larretxi I., Fernández-Gil M.d.P., Bustamante M.Á., Matias S., Navarro V., Simón E., Martínez O. (2022). Performance of Apple Pomace for Gluten-Free Bread Manufacture: Effect on Physicochemical Characteristics and Nutritional Value. Appl. Sci..

[33] Lidiková J., Čeryová N., Musilová J., Vollmannová A., Trebichalský P. (2025). Impact of Grape Pomace Addition on the Mineral Profile of Long-Life Bakery Products. Waste Forum.

[34] Tomar M., Bhardwaj R., Verma R., Singh S.P., Dahuja A., Krishnan V., Kansal R., Yadav V.K., Praveen S., Sachdev A. (2022). Interactome of millet-based food matrices: A review. Food Chem..

[35] Miller D.D. (2017). Minerals, in Fennema’s Food Chemistry.

[36] Ahuja A., Parmar D. (2017). Role of Minerals in Reproductive Health of Dairy Cattle: A Review. Int. J. Livest. Res..

[37] Haider A.M., Ali A., Saleh E.A.M., Jalil A.T., Abdulelah F.M., Romero-Parra R.M., Alkhayyat A.S. (2023). The Role of Chromium Supplementation in Cardiovascular Risk Factors: A Comprehensive Reviews of Putative Molecular Mechanisms. Heliyon.

[38] Demina E.N., Safronova O.V., Kuprina I.K., Kochieva I.V., Abaeva S.K. (2021). Research of the mineral composition of freeze-dried plant powders. IOP Conference Series: Earth and Environmental Science.

[39] Fiorentini D., Cappadone C., Farruggia G., Prata C. (2021). Magnesium: Biochemistry, Nutrition, Detection, and Social Impact of Diseases Linked to Its Deficiency. Nutrients.

[40] Gutzeit D., Winterhalter P., Jerz G. (2008). Nutritional Assessment of Processing Effects on Major and Trace Element Content in Sea Buckthorn Juice (*Hippophaë Rhamnoides* L. Ssp. Rhamnoides). J. Food Sci..

[41] Udensi U.K., Tchounwou P.B. (2017). Potassium Homeostasis, Oxidative Stress, and Human Disease. Int. J. Clin. Exp. Physiol..

